# SIGNATURE: A workbench for gene expression signature analysis

**DOI:** 10.1186/1471-2105-12-443

**Published:** 2011-11-14

**Authors:** Jeffrey T Chang, Michael L Gatza, Joseph E Lucas, William T Barry, Peyton Vaughn, Joseph R Nevins

**Affiliations:** 1 Department of Integrative Biology and Pharmacology University of Texas Health Science Center at Houston, Houston TX, USA; 2 Institute for Genome Sciences and Policy Duke University and Duke University Medical Center, Durham NC, USA; 3 Department of Biostatistics and Bioinformatics Duke University Medical Center, Durham NC, USA; 4 Department of Molecular Genetics and Microbiology Duke University Medical Center, Durham NC, USA

## Abstract

**Background:**

The biological phenotype of a cell, such as a characteristic visual image or behavior, reflects activities derived from the expression of collections of genes. As such, an ability to measure the expression of these genes provides an opportunity to develop more precise and varied sets of phenotypes. However, to use this approach requires computational methods that are difficult to implement and apply, and thus there is a critical need for intelligent software tools that can reduce the technical burden of the analysis. Tools for gene expression analyses are unusually difficult to implement in a user-friendly way because their application requires a combination of biological data curation, statistical computational methods, and database expertise.

**Results:**

We have developed SIGNATURE, a web-based resource that simplifies gene expression signature analysis by providing software, data, and protocols to perform the analysis successfully. This resource uses Bayesian methods for processing gene expression data coupled with a curated database of gene expression signatures, all carried out within a GenePattern web interface for easy use and access.

**Conclusions:**

SIGNATURE is available for public use at http://genepattern.genome.duke.edu/signature/.

## Background

Gene expression signatures are powerful tools that can reveal a range of biologically and clinically important characteristics of biological samples. In recent years, signatures have been developed that can differentiate distinct subtypes of tumors, identify important cellular responses to their environment (hypoxia), predict clinical outcomes in cancer, and model the activation of signaling pathways [1]. The power of gene expression signatures derives from their ability to connect an *in vitro *experimental state with an *in vivo *one in a quantitative manner. Commonly, the term gene expression signature has been used in two ways. In one, the signature is comprised of a set of genes that share a common pattern of expression. Sometimes this can be reported as genes that increase or decrease in expression, but the basic characteristic of the signature is the identity of the genes. Because of this, these signatures are often called *gene sets*. Gene sets have been curated from the literature and collected into databases such as MSigDB and GeneSigDB [2,3]. Tools have been developed that can analyze gene sets by looking for shared function or characteristics such as Gene Ontology terms [4] or drug sensitivity [5]. Another tool, single-sample GSEA has been previously applied to predict the co-regulation of gene sets from MSigDB on gene expression samples [6]. Evidence of co-regulation is then used to infer the activation of the phenotype embodied by the gene set.

The second type of signature relates the magnitude of increase or decrease in gene expression, in the form of weighted values, to a biological phenotype using a quantitative predictive model [6-16]. These signatures are often developed from experimental conditions that precisely control the phenotype of interest - for instance, the activation of a cell signaling pathway or the response of cells to a defined stimulus. Since the signature is comprised of a quantitative measure of the expression levels of genes that define the phenotype, it allows a direct measurement of the phenotype, rather than an indirect inference through co-regulation of genes in a gene set. A limitation of this approach, however, is the complexity of the methods used to derive and analyze the signatures, making it difficult to apply without significant multidisciplinary expertise [17].

Three major obstacles hinder the broad use of signatures. First, gene expression signature analysis requires the rigorous application of complex statistical methodologies on gene expression data. Second, it requires the acquisition and validation of data that properly capture the biological state of interest. Third, it requires a computational infrastructure that makes available the statistical software and data in an easy to use interface. In sum, gene expression signature analysis requires a confluence of expertise across a range of disciplines, including statistics, biology, and computer science.

While others have previously made use of our approach [16], it does require a level of expertise and computational infrastructure not always available in biological laboratories. This bioinformatic investigation, requiring the proper selection and application of statistical algorithms, as well as biological curation and validation of the signatures, can be daunting. Therefore, a challenge is how to develop software tools that enable such analyses for the general user. While it has long been recognized that software can target different types of users, a set of principles for software that is *biologist-friendly *was recently described [18]. In short, the recommendations are that the software 1) requires no knowledge of programming, 2) allows application of advanced methods, 3) can be used on different operating systems, and 4) provides a natural language description of the results. While such software has been developed for biological sequence alignment [19], sequence annotation [20], phylogenetic analysis [21], and comparison of prokaryotic genomes [22], no such platform exists for gene expression signature analysis. Because of this, and also because of the technical difficulty in performing gene expression analysis, we believe there is a need for a platform that captures a carefully refined analysis workflow, coupling algorithms and data, and enables a researcher to predict gene expression signatures on their samples.

## Implementation

To address the critical need for a platform for gene expression signature analysis, we have developed a collection of tools over the course of several years. First, we have developed BinReg, a statistical algorithm to predict the activation of a gene expression signature on a data set [23,24]. Second, we have curated a database of signatures that predict the activation of oncogenic pathways [25]. Now, we report on the development of a computational platform that combines these in a biologist-friendly interface, using the principles previously established. Here we describe the three components of a novel gene expression signature analysis platform, which we collectively call SIGNATURE.

### Component 1: The BinReg algorithm

**Figure 1:**
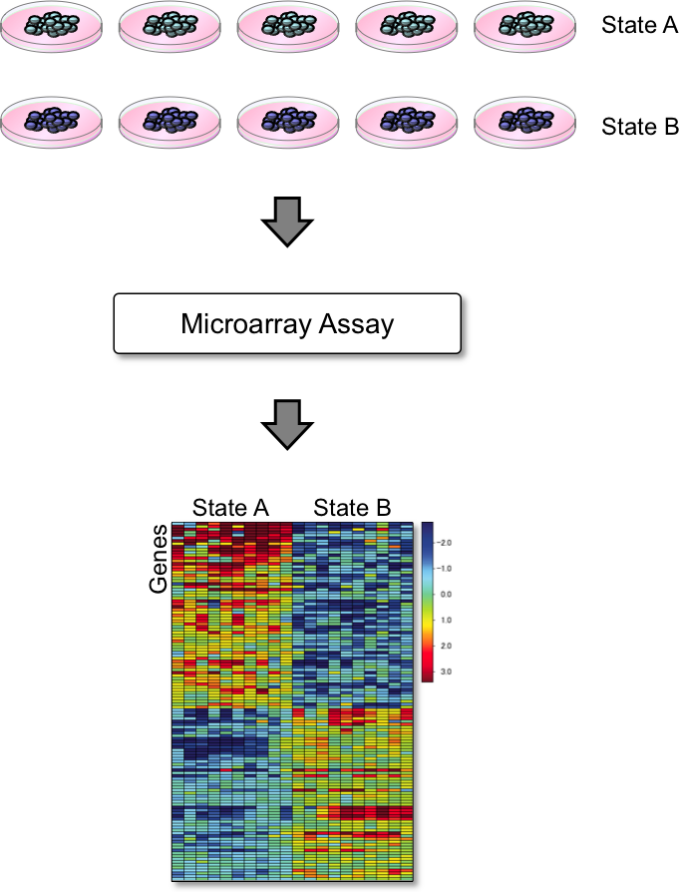
**Gene expression signatures**. Activation of biological processes, such as cell signaling pathways, results in a cascade of activities that ultimately lead to changes in the expression levels of genes downstream, the gene expression signature of that process. To measure that signature, we create experimental conditions that generate differential activation of the process in *in vitro *cell cultures. We then extract RNA from those samples for microarray analysis, which produces gene expression measurements from which we can score the signature. We show the gene expression signature in a heatmap where the rows are the genes that are differentially expressed in the process, and the columns are the samples that represent the two cellular states. Typically, multiple replicates are done. The colors indicate the expression levels of the genes, where the warm colors signify high expression and cool, low expression. This heatmap shows that the two states exhibit profoundly different gene expression patterns, allowing us to recognize the activation state of the pathway in a gene expression profile using a supervised machine learning algorithm.

The first component of SIGNATURE is the statistical analysis methodology. We frame gene expression signature analysis as a supervised machine learning problem. At its heart, a signature is a gene expression pattern that distinguishes two biological states (Figure 1). This might be the activation of a cell signaling pathway, the response of cells to various environmental inputs, or the intrinsic sensitivity or resistance of cells to a drug.

To create a signature for a given biological process, we first identify an appropriate training set consisting of gene expression data from samples that clearly distinguish the two desired characteristics (for instance, pathway on versus pathway off), called the train1 (on) and train0 (off) samples. To obtain the best distinction, these signatures are typically based on data collected from well-defined experimental perturbations. Using examples of cells in these two states, we select the genes whose expression levels best differentiate them based on a Pearson correlation. We then apply a Bayesian probit regression model to fit the genes in the training set to the two states. Because there are typically more genes than samples, we perform these computations in a reduced subspace. That is, we start with a gene expression data set *X *where *X *is a *p *× *n *matrix of signal values for *p *genes and *n *samples. We then factor *X *using a singular value decomposition such that *X *= *ADF*', where *A *is *p *× *k, D *is *k *× *k*, and *F *is *n *× *k*. *k *is the desired dimensionality of the subspace, which we call the number of *metagenes*. We create a model:

$$Y=\Phi \left({\left(A^{\prime }X\right)}^{\prime }\gamma \right)$$

where Ф is the cumulative density function of a normal distribution, *Y *is a vector of the posterior probabilities that the signature is active in each sample, and γ, the parameter to be sampled, is a *k *-dimensional vector of the contribution of each metagene. For the development of gene expression signatures, the number of metagenes chosen is a configurable parameter, where higher numbers of metagenes increase the complexity of the model, at the risk of potentially overfitting the training data.

The model is sampled using a standard Markov chain Monte Carlo algorithm. It produces the posterior probabilities *Y *as well as a 95% credible interval. *Y *should be interpreted as the probability that the pathway is active in each sample. The credible interval for *Y *indicates the upper and lower bound that can be set for the predictions, with 95% probability. Tighter bounds indicate higher confidence in the posterior probability *Y*, and wider ones indicate lower confidence. This statistical model has previously been described in detail [24].

Once a signature for a phenotype is developed, it can be used to score the phenotype in a new collection of samples. In all, a gene expression signature analysis requires seven parameters: 1 and 2) the train0 and train1 data, 3) the number of genes in the model, 4) the number of metagenes, 5) the algorithm used to preprocess the data set, 6) whether to apply quantile normalization, and 7) whether to apply shift-scale normalization. The first two parameters are the gene expression data that define the two cellular states. The next parameter specifies the number of genes to include in the statistical model. Then, the number of metagenes controls the complexity of the model [24]. For parameter five, we support two methods of preprocessing, RMA and MAS5 [26]. Parameters six and seven concern methods for normalizing the data to account for technical variation between the training and test sets. Quantile normalization has been described extensively in the literature. However, we use a variation of the algorithm whereby the quantiles are computed entirely from the training set to preserve independence between the training and test data. Finally, shift-scale normalization is an additional normalization method that, in short, adjusts the centroid and variance of the test set to match the training set.

### Component 2: A Database of Gene Expression Signatures

Over the past five years, we have developed and curated a collection of gene expression signatures that predict the activation of a large number of important cell signaling pathways, such as Ras, Myc, p53, and others [25]. Although this work has focused on developing signatures for pathways relevant to the study of cancer biology, the conceptual framework for this signature development is applicable across a wide range of other contexts. We envision that the current database would be most directly applicable to cancer studies, but there are also clear applications to other diseases with functional aberrations in these common pathways.

To simplify the analyses for general users, we determine empirically the best values for the seven parameters described above. Using a leave-one-out cross validation approach, we classify the samples in the training set. To ensure that the model is not over fit to artifacts or confounding factors in the original data, we then validate the selected parameters using an independent biochemical and/or genetic marker of pathway activity. The type of indicator used is specific to a pathway and depends on how it works. For example, to validate the PI3K signature, we compared against relative phosphorylated (active) p110 protein levels, and for the Estrogen Receptor (ER) pathway signature, the ER status in human breast tumors as determined by immuno-histochemistry [25].

Our signature database currently consists of 18 validated signatures, and we are actively developing and curating additional ones.

### Component 3A: Software tools for signature analysis

For gene expression signature analysis, we have developed software tools to cover two major use cases.

#### Use case 1: Predicting the activity of validated signatures

**Figure 2:**
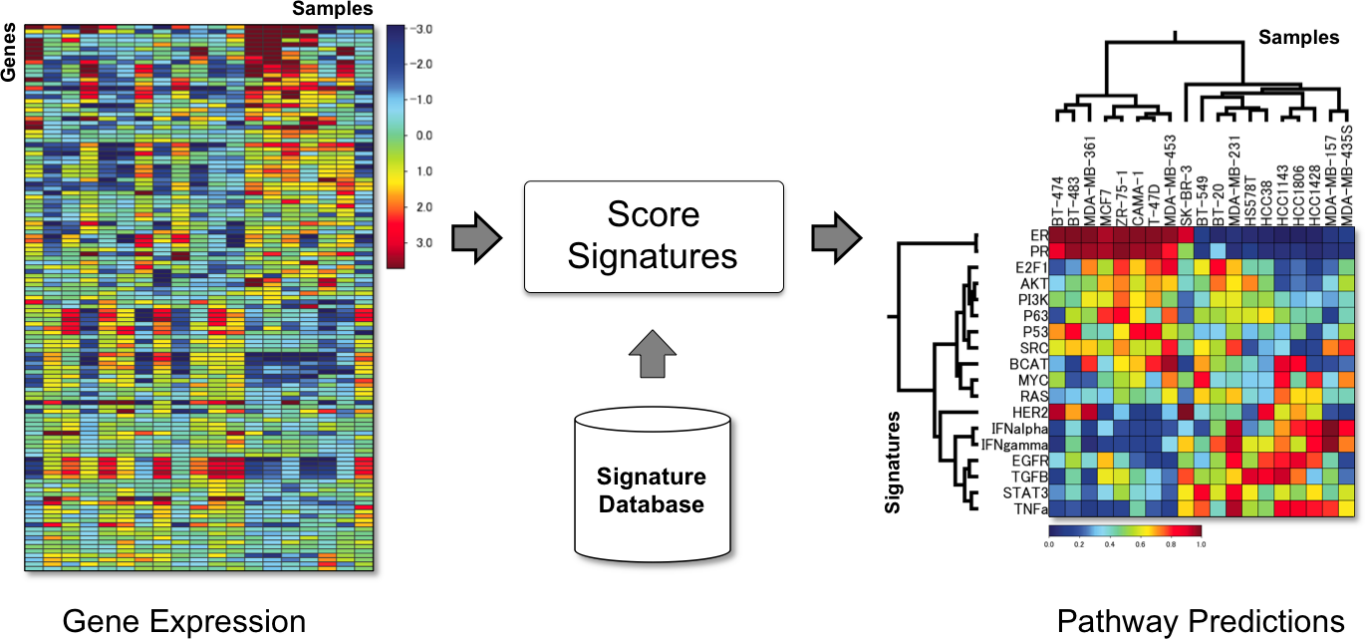
**The *Score Signatures *module**. This figure shows the workflow for the *Score Signatures *module. The user supplies a gene expression data set to study (shown on left). Then, this tool will retrieve a list of (currently 18) curated signatures from the signature database, and predict their activity in each of the samples in the data set (shown on right). The software also applies hierarchical clustering, showing the patterns of pathway activation. Here, *Score Signatures *was applied to a panel of 19 breast cancer cell lines. The clusters show that the signatures clearly distinguish two subtypes of breast cancer. In the left cluster are the cell lines of luminal origin. The right cluster consists of all basal cell lines, with two exceptions (SKBR3 and HCC1428). The module provides a biologist-friendly interface to a complex analysis that involves statistical algorithms and curated gene expression signatures.

Commonly, a user that has generated gene expression data from a set of experimental samples, such as a collection of human tumors, would wish to predict the activation of pathways in those samples. This user may not be familiar with the methodology to create signatures, or the computational algorithms to build a model of pathway activation. To address this case, we have developed a software application called *Score Signatures *that can apply the signatures from our curated signature database to a gene expression data set (Figure 2).

To use *Score Signatures*, one submits a gene expression data set of interest, such as that from a collection of tumor samples. The application will then apply our Bayesian algorithm to predict the activation of the signatures in the database. The output is a series of probability scores for each signature, reflecting the extent to which the signature is represented in each sample from the test data set. These probability scores are depicted in a heatmap that shows the pattern of activation of the pathways across the data set as determined by hierarchical clustering. Furthermore, *Score Signatures *also provides raw data as tab-delimited text files that can be accessed with standard tools such as Microsoft Excel and used to develop additional plots. These results are summarized in a human-readable report with a detailed description of the analysis as well as guidelines for interpreting the results.

Each *Score Signatures *analysis is comprised of Bayesian regression calculations that predict the activation of each signature from the signature database. A full analysis is described using a large number of parameters, seven for each pathway in the database. The challenge here is how to provide the analyses so that it is accessible for users that are not familiar with the technical details of gene expression analysis. We solve this issue by storing the validated parameters in the database. As a default, the values are retrieved from the signature database, ensuring that the signature runs in precisely the manner originally defined. However, for expert users, we make it possible to refine each parameter, and if changed, the system will document the deviation from the default. In this way, the needs of both general and expert users can be met.

#### Use case 2: Developing a novel signature

*Score Signatures *provides a convenient way to apply the signatures from our signature database on a data set. However, it does not have an ability to generate a new signature. To address this, we have produced a second application, *Create Signature*, to develop novel gene expression signatures.

While *Score Signatures *can be used by investigators with little or no knowledge of the details of the underlying methodology, *Create Signature *requires an understanding of the machine learning framework and the parameters used to create the signatures. The user specifies the values for a total of 15 parameters. In addition to the seven parameters for the signatures as described above, it also includes parameters that govern the MCMC simulation of the Bayesian model, and others (such as other normalization methods) that we have not used in our signatures.

Once the parameters are specified, *Create Signature *generates a statistical model from the training set and predicts signature activity in both the training set (using leave-one-out cross validation) and the test set (after building a model from the entire training set). Similar to *Score Signatures, Create Signatures *also provides publication-ready plots, raw data, and a human-readable report of the key results, fulfilling a critical requirement of user-friendly software described above.

### Component 3B: A web interface for gene expression signature analysis

Our analysis tools are delivered through GenePattern [27]. The GenePattern platform provides a web-based interface for external programs (or *modules *in GenePattern terminology) via a plug-in architecture. However, one limitation with GenePattern is that it does not have the means to provide a context-dependent interface that *Score Signatures *requires. That is, the interface for *Score Signatures *depends on the current state of the signature database, as well as the requirements of the user. *Score Signatures *requires (currently) a total of 74 parameters, but only two are likely to be changed by the vast majority of users. In this situation, the system needs the facility to hide rarely used parameters for novice users, but allow advanced users to tune them. This is not currently possible in GenePattern.

To address the limitations of GenePattern, we have extended GenePattern with an *interface generator *layer. An interface generator is an optional component of a module that is responsible for defining its interface, that is, the parameters that are provided for the user. This is implemented by modifying the GenePattern source code so that when a user accesses a module, GenePattern can retrieve the interface from the interface generator instead of its own default mechanism. Technically, interface generators are CGI scripts, which provide them the ability to access external resources, such as the signature database.

**Figure 3:**
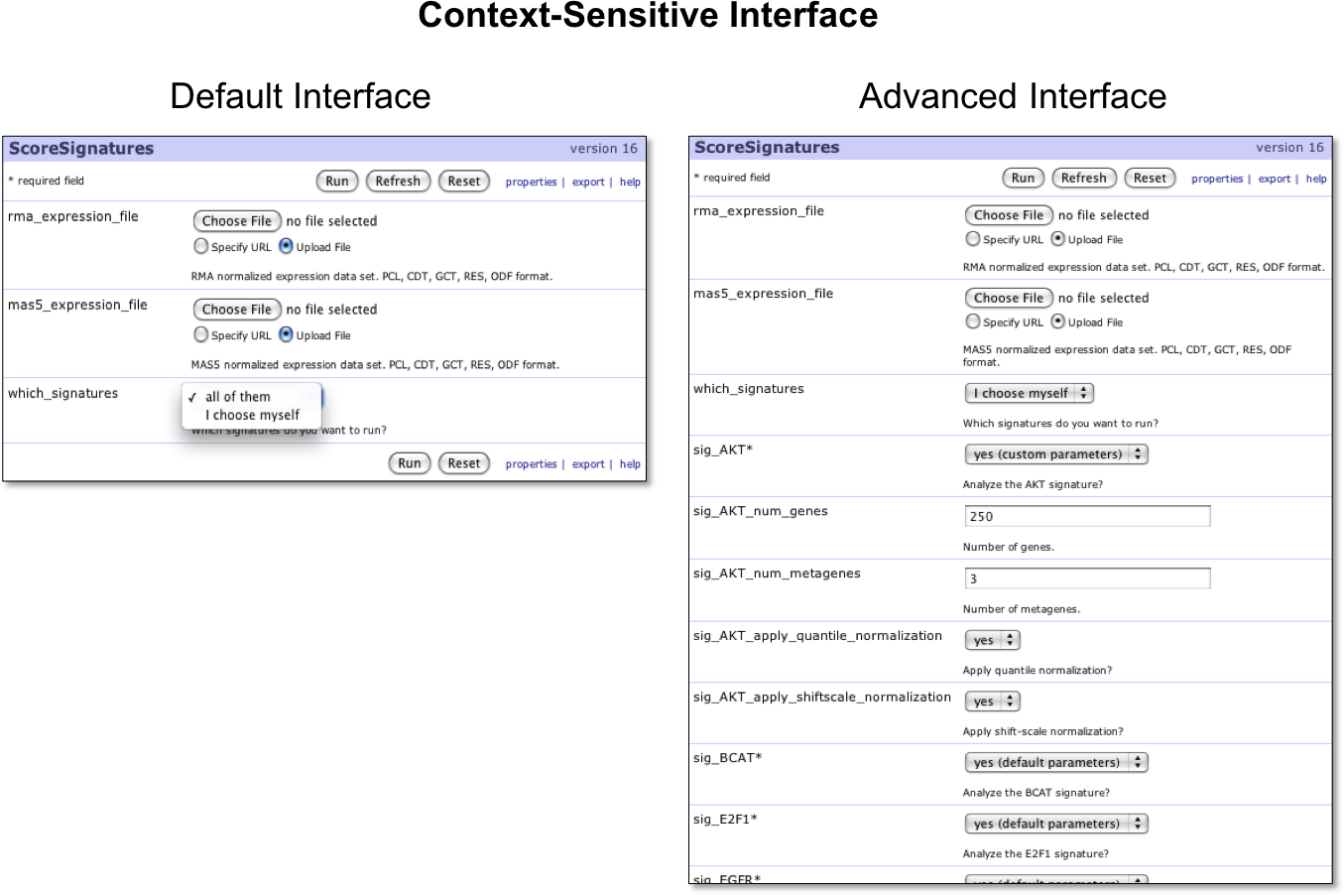
**The SIGNATURE interface in GenePattern is context-sensitive**. To develop interfaces that can be sensitive to context, such as the state of a database or expertise of the user, we have extended GenePattern to support Interface Generators. These are add-ons to standard GenePattern modules that are responsible for producing the interface for the module at run time. Interface Generators can serve as brokers between GenePattern and other databases across the network. An Interface Generator is used for the *Score Signatures *module. In the simplest form of a *Score Signatures *analysis, the only parameters necessary are the RMA and MAS5 normalized versions of the data set, and an option for which signatures to include in the analysis (left). However, experienced users may wish to tune the analysis, and in response, the interface presents the user with an advanced interface that allows tuning of every parameter. On the right, a user has chosen to tune the parameters for the AKT signature.

**Figure 4:**
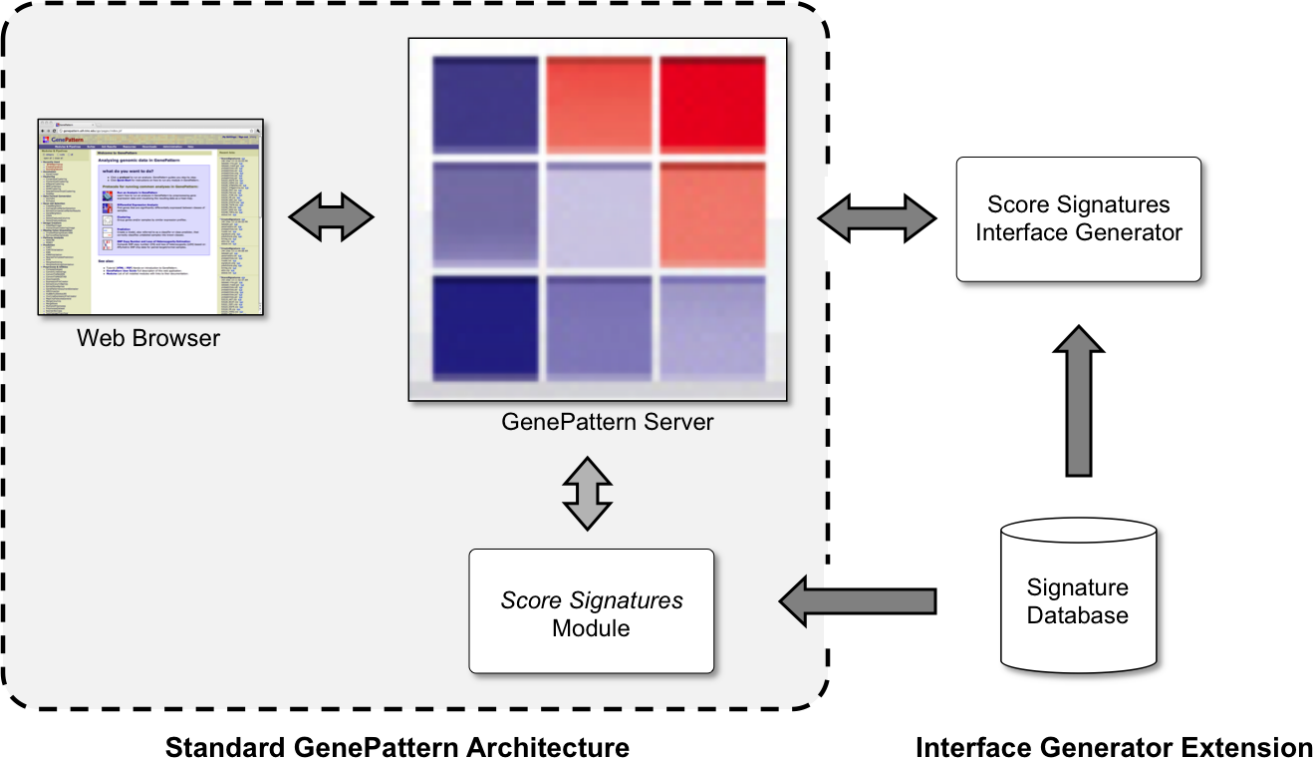
**Implementation of an Interface Generator**. The GenePattern server provides a web-based interface for modules that perform bioinformatics analyses. Because the parameters for a *Score Signatures *analysis change frequently with updates to the Signature Database, the interface for *Score Signatures *cannot be defined until run-time. To address this, we have extended GenePattern so that it can create the interface for modules dynamically by querying an interface generator. The interface generator for *Score Signatures *queries the signature database at run-time to produce an up-to-date interface for GenePattern. Once the user has specified their values for the parameters, GenePattern invokes the *Score Signatures *module to perform the analysis. This is a general mechanism that can be applied to other complex analyses.

The interface generator for the *Score Signatures *module, by default, creates an interface with only three parameters: the RMA-formatted gene expression file, the MAS5-formatted gene expression file, and a checkbox to show advanced options (Figure 3). If the user chooses to activate the advanced interface, GenePattern makes another request to the Score Signatures interface generator, which then produces an interface that includes parameters that are retrieved in real time from the Signature Database (Figure 4). In this way, GenePattern can provide an interface that is responsive to the needs of the user as well as the current status of other data resources. By having such an ability, we can deploy context-sensitive interfaces whose complexity matches the needs of the user.

## Results

We have developed a public software platform SIGNATURE that simplifies gene expression signature analysis by providing an easy to use GenePattern interface on top of a complex infrastructure of analysis software and a signature database. Specifically, we have developed BinReg, a Bayesian probit regression algorithm that has been supplemented with metagenes and normalization functions to handle the idiosyncrasies of gene expression data. Also, we have curated and validated a database of 18 gene expression signatures for activated oncogenes. And finally, we have significantly extended GenePattern by developing an interface generator layer that can produce context-sensitive interfaces to fit the needs of the user.

One limitation of SIGNATURE is that the predictions are dependent upon the quality of the data. One potential factor that can confound the interpretation of the results is the presence of batch effects or other technical variation after the applied normalization [28]. In our experience, we have observed that technical artifacts lead to broad changes in the expression profiles that lead to homogeneous predictions. That is, the predicted scores tend to cluster around the same probability, typically around 0% or 100%. This issue highlights the fact that these predictions should be confirmed with alternate assays. Currently, the tools available within SIGNATURE require expression profiles to be annotated with probe sets from Affymetrix U133 microarrays. To apply them to microarrays from other platforms, the probes would need to be converted to these U133 probe sets. Internally, we have successfully applied SIGNATURE to gene expression data from Illumina BeadArrays (data not shown), suggesting a high degree of reproducibility in the gene expression levels between these two platforms, consistent with prior reports [29,30]. However, we have had more limited success in converting signals from cDNA arrays, and have not tried applying these analyses to expression data from sequencing platforms. We believe the ability to apply these methods depends on the reproducibility of the expression signals across platforms.

## Conclusions

In conclusion, the SIGNATURE platform comprises two modules, *Score Signatures *and *Create Signature*, that are most widely useful in interpreting gene expression data. However, we have also created modules for more specialized analyses that we have previously described (Table 1). SIGNATURE provides a general framework that can be used to deliver complex algorithms in a user-friendly manner, putting sophisticated bioinformatic analyses, such as gene expression signature analysis, within reach of a larger audience.

**Table 1:** Modules available in SIGNATURES

Module	Use Case	Publication
ScoreSignatures	To predict activation of pathways in gene expression data.	
CreateSignature	To create a new gene expression signature using a training set.	1
BFRMNormalize	To remove technical variation across one or more gene expression data sets.	2
FindSubtypes	To find subtypes within a gene expression data set.	3
PredictSubtypes	To assign a subtype to gene expression data using a previously developed model.	3
BFRMFactor	To dissect a gene expression data set into modules.	4
BFRMProject	To score the modules in gene expression data using a previously developed model.	5

SIGNATURE includes modules that perform a range of analyses on gene expression data ^1^[24]^2^[31]^3^[25]^4^[32]^5^[33].

## Availability and requirements

SIGNATURE is available for public use, without need for a material transfer agreement, at http://genepattern.genome.duke.edu/signature/. This page includes a link to the modules available on GenePattern, as well as sample data for testing purposes. The source code and gene expression signature database are also available from this page.

Project name: SIGNATURE

Project home page: http://genepattern.genome.duke.edu/signature/

Operating system: platform independent

Programming language: Python, C, R, Matlab

Other requirements: web browser

License: MIT

Any restrictions to use by non-academics: none

## Authors' contributions

JTC and JRN conceived of the project. MLG developed the gene expression signatures. JTC, JEL, WTB, and PV developed the software. JTC and JRN wrote the manuscript. All authors have read and approved the final manuscript.
